# A Concurrent and Hierarchy Target Learning Architecture for Classification in SAR Application

**DOI:** 10.3390/s18103218

**Published:** 2018-09-24

**Authors:** Mohamed Touafria, Qiang Yang

**Affiliations:** 1 Department of Electronic Engineering, Harbin Institute of Technology, Harbin 150001, China; 2 Collaborative Innovation Center of Information Sensing and Understanding, Harbin Institute of Technology, Harbin 150001, China

**Keywords:** Automatic Target Recognition (ATR), Synthetic Aperture Radar (SAR), Convolutional Neural Networks (CNN), Fisher Vectors (FVs), Moving and Stationary Target Acquisition and Recognition (MSTAR)

## Abstract

This article discusses the issue of Automatic Target Recognition (ATR) on Synthetic Aperture Radar (SAR) images. Through learning the hierarchy of features automatically from a massive amount of training data, learning networks such as Convolutional Neural Networks (CNN) has recently achieved state-of-the-art results in many tasks. To extract better features about SAR targets, and to obtain better accuracies, a new framework is proposed: First, three CNN models based on different convolution and pooling kernel sizes are proposed. Second, they are applied simultaneously on the SAR images to generate image features via extracting CNN features from different layers in two scenarios. In the first scenario, the activation vectors obtained from fully connected layers are considered as the final image features; in the second scenario, dense features are extracted from the last convolutional layer and then encoded into global image features through one of the commonly used feature coding approaches, which is Fisher Vectors (FVs). Finally, different combination and fusion approaches between the two sets of experiments are considered to construct the final representation of the SAR images for final classification. Extensive experiments on the Moving and Stationary Target Acquisition and Recognition (MSTAR) dataset are conducted. Experimental results prove the capability of the proposed method, as compared to several state-of-the-art methods.

## 1. Introduction

The classification of Synthetic Aperture Radar (SAR) targets into different classes is one of the most challenging algorithmic aspects of radar structures. Unlike optical remote sensing, which cannot achieve its role in bad weather and at night, SAR can operate in all-weather conditions day-and-night and make very high resolution images, and it has played an important role in military and civil applications, such as target classification, reconnaissance, and surveillance. However, the comprehension of SAR images requires specialists, because unlike natural images, SAR images reflect the backscattering electromagnetic wave intensity of targets and speckle. Moreover, for humans, searching for targets of interest in the massive SAR images will take a lot of time. Furthermore, SAR images are covered with speckle noise, which is an important reason behind the reduction of the images quality. Besides this, they are very sensitive to the variation of target pose, and vary suddenly and quickly with small change in aspect angles. Therefore, SAR Automatic Target Recognition (ATR) is a demanding mission and has become a serious research topic for many applications. In the last decade, several methods were proposed in order to analyze and classify SAR images. A basic architecture of SAR ATR was defined as three phases: Detection, discrimination, and classification [1]. The primary two stages, also known as prescreening and low-level classification (LLC), are ordinarily referred to as the focus-of-attention module [2]. Detection is to excerpt candidate targets from SAR images via a Constant False Alarm Rate (CFAR) detector [3]. The output includes targets of interests such as armored vehicles, missile launchers, and tanks as well as false alarm clutter such as buildings, bridges, trees, and cars. In order to dispose of false alarms, several features are chosen to train a discriminator to distinguish between the two classes (target and clutter), which is the discrimination stage [4]. The third stage, also called high-level classification (HLC) [5], aims to classify the targets into different categories, which is the focus of this research.

Of the numerous methods, each approach that has been proposed to deal with the classification stage is included in one of the three main taxonomies, namely template-based [6], model-based [7] and pattern-based [8]. In recent years, pattern-based algorithms have made significant breakthroughs in the image classification process. A variety of architectures have been proposed, and they are based on first designing a set of different features to represent the targets by extracting them directly from the raw images, and then using these feature vectors to train a classifier and hence the classification problem is solved. To obtain a suitable classification performance, the features together with the classifiers should be carefully constructed. On one hand, numerous algorithms are employed for feature extraction, for instance, scale invariant feature transform (SIFT) [9], histogram of oriented gradients (HOG) [10], non-negative Matrix Factorization (NMF) [11,12], Principal Component Analysis (PCA) [13,14], deep Convolutional Neural Networks (CNNs) [15,16]. On the other hand, several robust trainable classifiers have been developed such as mean square error (MSE) classifier [17], Bayes classifier [18] and support vector machine (SVM) [19].

Deep CNN is currently one of the most promising technologies for classification. It has obtained the state-of-the-art results for object detection and automated land cover classification in high-resolution remote-sensing imagery. Deep CNN has been used to classify a wide variety of remotely sensed imagery, including SAR images [20], hyperspectral [21], and high resolution electro-optical imagery [22]. Different from methods based on hand-crafted feature extraction, the deep CNN based method automatically learns the feature from large-scale datasets, and achieves extraordinary performance in object recognition. The goal of CNN is to catch exceptional representations, usually at various levels, using the unknown structure in the input data, where lower-level features make it possible to define and learn higher-level features and make them more abstract, with their individual features. Then, these discovered features are more invariant to most of the variations commonly present in the raw training distribution, while collectively conserving the essential information in the input. Taking the tremendous progress deep learning has made in object recognition into consideration, deep CNNs are expected to also solve the SAR target recognition issue. Several researchers replicate architectures of deep CNN, which were successfully applied on object classification on SAR images. However, they did not obtain the same results because training a deep CNN requires a very large dataset such as ImageNet [23,24,25,26], which is clearly not the case of SAR images. Nonetheless, the limited labeled SAR target data have not stopped researchers from achieving better classification rates. To address this problem, numerous approaches have been employed to achieve better results on SAR images like transfer learning and training CNN from scratch.

One technique consists of applying transfer learning from pre-trained deep CNNs that can be modified to produce accurate classifications for specific applications. The main idea of CNN transfer learning is to use the power of networks that are already trained on a large-scale data set of general images in different application areas. These types of networks can be regarded as general feature extractors or classifiers. This technique can be successfully applied in cases where we do not have a large data set for training a CNN from scratch. There are three famous scenarios for transfer learning of a CNN: A pre-trained net is used as a fixed feature extractor, a pre-trained net is used for fine-tuning and a pre-trained net is used for initialization in training from scratch [27,28,29]. In the first approach, a neural network is trained with an independent general data set of images and the output of the network can be interpreted as a feature vector and used for further classification. In addition, features from the previous to last hidden layer can be used for classification. The fine-tuning method is based on the idea of training the last layers of the network to specialize them for a particular data set. The main benefits of this method are reduced training time and the possibility of effective training with a small data set. However, this technique does not perform well on SAR images since they refer to the backscattering characteristics of the ground features, representing a list of scattering centers, and each pixel intensity of the image depends on a range of factors, such as shapes, orientations and types of the scatterers in the area where the target is located. Other techniques were proposed to overcome this problem, one of which consist of training a CNN from scratch using data augmentation to increase the size of data [20]. Another method uses transfer learning from pre-trained deep CNN. However, instead of using optical images data set in the training, it uses a large number of unlabeled SAR scene images [27].

Another interesting point that is worth noting is the use of feature coding approaches in the process of images classification. The Bag-of-Words (BoW) [30] approach is one of the famous models in this field. In order to have good classification accuracy, three steps were performed: First extract the features, then generate a codebook, and after that a histogram is generated to represent each image. Many modern approaches are based on the BoW model; for example Reference [31] proposed a supervised incremental coding method based on the BoW model and proved that this method yielded much better features for SAR image classification. One recent method uses Fisher Vectors (FVs), which are in essence an image representation obtained by pooling local image features, and they are used as a global image descriptor in image classification. Compared to the previous coding approaches, its advantages are: First, its ability to store second-order information about the features. Secondly, FVs utilize Gaussian Mixture Models (GMMs) to generate the feature vocabulary. Therefore, it generates a probabilistic visual vocabulary instead of using a hard codebook, which allows it to be more flexible. This is an important feature, which helps in increasing the accuracy performance.

Another important technique that can be utilized to improve classification accuracy is fusion. Sensor fusion is a common technique in signal processing to combine data from various sensors. Feature fusion is another current method, ranging from simple concatenation to very advanced methods like fuzzy integrals. Finally, information fusion merges independent results from signal processing techniques that otherwise can be used alone as the final signal processing result. For example, Reference [32] has obtained good results by using some of these techniques on Land Cover High-Resolution Imagery.

The CNN architectures proposed in the literature have proven that the activations from high-level layers of CNNs can generate powerful feature representations with outstanding performance. However, we noticed that the best classification accuracy for individual SAR classes varied among the different CNN architectures. In addition, features extracted from lower-level layers, particularly the convolutional layers, lack sufficient study. Only a few works has been conducted in this area; for example, Reference [33] used cross-layer CNN features extracted from multiple layers of CNN for generic classification tasks. Reference [34] on the other hand has used features extracted from the last convolutional layer of deep CNN using transfer learning for the scene classification of high-Resolution remote sensing images. They both demonstrate the capacity of lower-level layers of CNN architectures to achieve better performance.

The two reasons mentioned above encourage us to propose a new framework. The framework is drawn from the most contemporary techniques in image processing and deep learning. It is a combination of three different CNNs. The three CNNs have the same architecture; the main difference is the sizes of the convolution and pooling kernel in each one of them: Coarse Grain CNN (CG-CNN), Middle Grain CNN (MG-CNN) and Fine Grain CNN (FG-CNN). We train each CNN from scratch using the chosen sizes of convolutional and pooling kernel. We show that through a combination of recent techniques, we can obtain significant performance improvement on the Moving and Stationary Target Acquisition and Recognition (MSTAR) dataset classification task. We investigate how to obtain better accuracy classification compared to the state-of-the-art results that use the same dataset without data augmentation by forming better representations from the SAR images using CNNs activations. By deleting the last few layers of a CNN, we handle the remainder of the CNN as a fixed feature extractor. Considering that these CNNs are broad multi-layer architectures, we consider two ways of extracting CNN features with reference to different layers:

- We simply calculate the CNN activations for the entire SAR images and consider the Fully-Connected (FC) layer activation vectors as the global feature representations for all images.
- We first compute dense CNN activations from the last convolutional layer of the input image, and then we convert them into a global representation using the Fisher encoding. Then, the global features of the image are fed to a simple classifier for the classification task.

Extensive experiments prove that powerful features SAR images can be generated. The resulting features and classification outputs from each of these CNNs are then either combined or fused using a variety of methods into a final refined classification. Evaluation of the proposed approach is conducted with the MSTAR benchmark data set. Experimental results validate the superiority and effectiveness of the proposed approach.

The paper is formulated as follows. In Section 2, we briefly review some related works corresponding to some state-of-the-art SAR images classification methods. Section 3 describes the main methods and tools used in this work, followed by the fusion method used as well as the fuse classifiers for SAR images classification. Section 4 presents the proposed framework used to extract, process, and classify the features. In Section 5, experiments are carried out with the MSTAR database, and the performance of the proposed approach is described. Finally, Section 6 lists the conclusions along with the discussion of the results.

## 2. Related Work

In this section, a quick overview of the previous studies related to SAR target recognition is given. Cui Z [35] reviewed the effectiveness of some traditional methods such as PCA and NMF, which have an accuracy of 86.07% and 89.47%, respectively. These traditional methods have a relatively low accuracy rate compared with recent approaches. Agrawal and Mangalraj [36] proposed a feature extraction algorithm based on SIFT and they achieved an accuracy of 90.99% in a 3-class classification task.

Over the last decade, many researchers have focused on the classification of SAR ATR using CNN, which normally refers to supervised classification. Using randomly sampled SAR targets patches, Reference [30] extracts SAR targets feature representation by a single convolutional layer and achieves the accuracy of 84.7% in 10-class classification tasks. Reference [16] learned discriminative feature sets directly from training data instead of requiring pre-specification by a human designer. In order to achieve that the author used an architecture of three convolutional layers, followed by an FC layer of Softmax as a classifier, and recorded an accuracy of 92.3%. Since then, various learning algorithms were explored such as AdaDelta [35], which can update the different learning rates of hyper-parameters and outperformed other methods like stochastic gradient descent (SGD) and AdaGrad. Reference [27] proposed a framework based on transfer learning where an assembled CNN architecture consisting of a classification pathway and a reconstruction pathway were designed. The author used a large number of unlabeled SAR scene images to train the reconstruction pathway using stacked convolutional auto-encoders instead of training the CNN with the limited dataset. The results showed an accuracy of 99.05%. Reference [20] proposed an architecture of five-layer all-convolutional layers (A-ConvNets) using the data augmentation technique to enlarge the dataset, since the limited training data was insufficient to train the deep CNN, and used two techniques to avoid over-fitting which are removing the FC layer and a drop-out in a convolution layer. The experimental results illustrate that A-ConvNets achieved an average accuracy of 99% on the classification of ten-class targets.

## 3. Methods and Materials

### 3.1. Deep Convolutional Neural Networks (CNNs)

**Figure 1:**
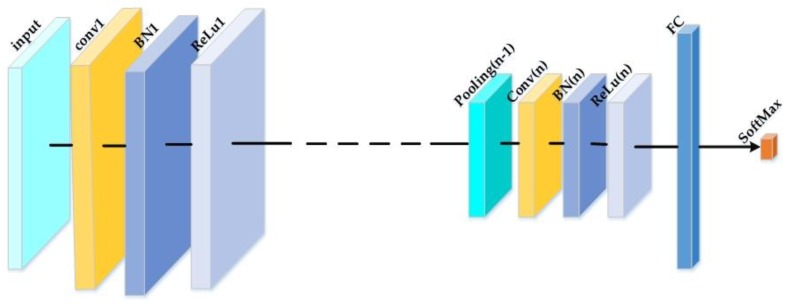
Typical architecture of a CNN.

Deep CNNs are neural networks whose topology encodes a spatial relationship between nodes in successive layers that is analogous to the convolution operation. A deep CNN is an algorithm, which learns a nonlinear classification function from the labeled input data. It is characterized in this way: First, an input layer, where the algorithm receives the SAR images of the MSTAR dataset and transfer them to the next layers for further processing. Next, a number of hidden layers follows the input layer. The role of hidden layers is to perform feature extraction and aggregation from the raw data. In a typical CNN, the hidden layers are alternating sequences of convolutional, batch normalization, ReLu and pooling layers. Finally, the architecture has an output layer that acts as a classification mechanism. Each layer has neurons that act upon the input features and pass the result to the next layer. An effective network learns weights in each layer, which produce accurate classification outputs for their input images. When training a deep neural network, the main challenges are in setting up the correct topology, choosing appropriate parametric initializations, defining an objective function to quantify misclassification error and an appropriate algorithm for training. All these factors affect the performance of the network. A multitude of extensions to the basic architecture and training mechanism have been proposed, the most recent one which have better results is composed of multiple cascaded stages, as is shown in Figure 1.

The convolutional layer’s outputs are feature maps; each one is computed by a dot product between the local regions (receptive field). Training CNN is complicated since the distribution of each layer’s inputs changes during training, as the parameters of the previous layers change (internal covariate shift). This slows down the training by requiring lower learning rates and careful parameters initialization, and makes it notoriously hard to train models with saturating nonlinearities. Therefore, a Batch Normalization (BN) layer is added before the nonlinearity where elements of feature maps, at different locations, are normalized. The BN layer’s role is to reduce the internal covariant shift and the gradients dependence on their initial values or on the scale of the parameters. It also reduces the need for dropout and regularizes the model [37]. Principally, an elementwise non-linear activation function is practiced to these feature maps like ReLu or sigmoid. By computing a local region maximum, the max-pooling layers complete a down sampling operation on the feature maps spatial dimensions. The FC layer has full connections to the totality of activations in the previous layer, and their activations are computed with a matrix multiplication followed by a bias offset. The last layer is a Softmax layer that computes the scores for each defined class. CNNs transform the input from original values to the final class scores through the structure in a feedforward style. The CNNs parameters (i.e., the weights and bias) are trained with classic stochastic gradient descent with momentum based on the backpropagation algorithm [38,39].

### 3.2. Feature Coding

Feature coding is a key component in image classification. It has been extensively studied over the past decade, where numerous coding algorithms have been proposed. The bag-of-features (BoF) [40], developed from the BoW model in document analysis [30], is one of the most popular and effective image classification frameworks in the recent literature. It has achieved impressive performance in several databases and competitions. Another method, which can be viewed as a generalization of the BoW, is the Fisher coding-based method, which has gained popularity in the last few years. Due to its special characteristics compared with the other image representation methods, it is well suited for SAR images, as FVs method is based on a visual vocabulary, with the assignment of patches to visual words and can extract a larger image signature for a given number of visual words than other representations such as the BoW. Moreover, the FVs record also the mean and variance of the points per dimension in each cell, which means having more information for same visual words. Therefore, compared with the other methods the FVs lead to high-dimensional feature vectors. In addition, FVs can be computed from much smaller vocabularies, and therefore at a lower computational cost. FVs are designed to estimate the probability density distribution (PDD) of features and focus on the global description of all features, achieving translation and rotation-invariance, which is crucial to classifying SAR images.

#### Fisher Vectors

FVs coding-based methods [41,42] estimate the distribution of features with the GMM, consisting of the weights, the means, and the covariance matrix of multiple Gaussian distributions, each of which reflects one pattern of features. FVs coding was originally derived from the Fisher kernel, which aims to combine the benefit of generative and discriminative approaches for pattern recognition by describing a signal by a gradient vector from its probability density function [43]. The gradient vector indicates the direction in which parameters should be adjusted to best fit the data.

Generally, FVs coding process can be divided into front-end stage and back-end stage. In our case, the front-end stage consists of extracting features from the last convolutional layer. The back-end stage employ GMM as the generative model for these local features. The FVs are then derived from the gradients with respect to the GMM parameters. Most existing methods employ a diagonal GMM as a generative model to represent the distribution of local features, for instance SIFT [9] and HoG [10]. Several versions of Fisher coding have been proposed after the original one [41]. As far as we can tell, Improved Fisher Kernel(IFK) [42] achieves the best performance. 

Let $X=\{x_{n},n=1\ldots N\}\in R^{D\times N}$ be the features extracted from an image in a D-dimensional descriptor space, $B=\{b_{m},m=1\ldots M\}\in R^{D\times M}$ be a codebook with M codewords (obtained typically by clustering over features), and $V=\{v_{n},n=1\ldots N\}$ be the corresponding representation of these N features. Each *x* is represented by the codebook *B* in feature coding. This process generates responses on M codewords, consisting of a coding vector *v* with M elements. In most coding algorithms, only a part of codewords is chosen to represent a feature, and therefore the coding vector *v* is usually sparse, i.e., the responses are zeros on the codewords which are not chosen. The GMMs define the probability density distribution of features in IFK. Its parameters $\theta_{n}=\{\alpha_{n},\mu_{n},\sigma_{n}\}$ are the weight $\alpha_{n}$, the mean vector $\mu_{n}$, and the covariance matrix $\sigma_{n}$ of the *n*th Gaussian distribution can be generally estimated by the Expectation Maximization (EM) algorithm [44]. Supposing features are independent from each other, each image can be expressed by the log likelihood of all extracted features:

(1) $$L(X/\theta )=\sum_{n=1}^{N}\log p(x_{n}/\theta )\;$$

where $p(x_{n}/\theta )$ is the GMM-based probability density function. The normalized gradient vector is represented as:

(2) $$\Gamma =F_{\theta }^{-1/2}\nabla_{\theta }L(X/\theta )\;$$

(3) $$\nabla_{\theta }L(X/\theta )=[\frac{\delta L}{\delta \alpha },\frac{\delta L}{\delta \mu },\frac{\delta L}{\delta \sigma }]$$

The derivative to α has very little contribution to the performance according to Reference [42]. Thus, it is removed in IFK.

(4) $$\nabla_{\theta }L(X/\theta )\approx [\frac{\delta L}{\delta \mu },\frac{\delta L}{\delta \sigma }]\;$$

(5) $$F_{\theta }=E_{X_{p},X_{q}}[\nabla_{\theta }L(X_{p}/\theta )\nabla_{\theta }L(X_{q}/\theta )]\;$$

where $F_{\theta }$ is the Fisher information matrix, $X_{p}$ and $X_{q}$ denote two sets of features extracted from two arbitrary images. The Fisher information has an approximated close solution according to References [42,45], with which the coding vector of a feature, i.e., the FVs, can be represented as:

(6) $$v(k)=[\Gamma_{\mu ,k},\;\Gamma_{\sigma ,k}],k=1,\ldots ,M\;$$

(7) $$\Gamma_{\alpha_{k}}^{X}=\frac{1}{\sqrt{w_{k}}}\sum_{n=1}^{N}\gamma_{n}(k)\left(\frac{x_{n}-\mu_{k}}{\sigma_{k}}\right)$$

(8) $$\Gamma_{{}_{\sigma_{k}}}^{X}=\frac{1}{\sqrt{w_{k}}}\sum_{n=1}^{N}\gamma_{n}(k)\frac{1}{\sqrt{2}}\left(\frac{{\left(x_{n}-\mu_{k}\right)}^{2}}{\sigma_{k}{}^{2}}-1\right)\;$$

(9) $$\gamma_{n,k}=w_{k}p_{k}(x_{n}/\theta )/\sum_{j=1}^{M}w_{j}p_{j}(x_{n}/\theta )\;$$

The FVs are obtained via quantizing the set of local feature descriptors with a small codebook and aggregating first and second order residual statistics for features quantized to each centroid. In other words, the fisher encoding captures the average first and second order differences between the image descriptors and the centers of a GMM, which can be thought of as a soft visual vocabulary. The residual statistics of centroids are concatenated together to attain the high-dimensional global descriptor representation. The performance has a proportional relation with the dimensionality of the global descriptor as shown in Reference [46]. 

FVs can be aggregated on descriptors in the image [45] which is popular for image classification, or around interest points [47] which are invariant to scale and orientation. The latter is usually used in image retrieval as the Difference of Gaussian interest points provide invariance to scale and rotation, which make FVs in image retrieval more invariant to certain geometrical transformations of the images and robust to training data [48].

### 3.3. Information Fusion

In this section, we focus on information fusion of the CNN classifier outputs to obtain improved results for the classification of the MSTAR data sets. The techniques summarized in the following are used in this paper to fuse the different CNN architectures outputs to obtain the final classification result. In our subsequent classifier fusion discussion, we use the following notation:

Suppose that we have a set of K classifiers, each of which classifies targets into one of distinct M classes. The output vector of classifier *c_k_*, given a target X, is represented by a column vector.

(10) $$y_{k}=\{y_{k,m};m=1,2,\ldots ,M\},where:k=1,2,\ldots ,K\;$$

where *m* is the component of the output vector *y_k,m_* represents the estimated posterior probability that target X belongs to the class *m*, estimated by classifier *c_k_*. *y_k,m_* satisfies the following two requirements:

(11) $$0\leq y_{k,m}\leq 1$$

(12) $$\sum_{m=1}^{M}y_{k,m}=1$$

The classification decision of classifier *θ_k_* is:

(13) $$\theta_{k}=\arg\underset{1\leq m\leq M}{\max}\{y_{k,m}\}$$

C is the classification matrix of size K×M, corresponding to K classifiers for *M* classes; $p_{k}$ is per-class classification accuracy from the CNN, for k∈[1,…,K] calculated for each network as follows:

(14) $$p_{k}=[\left(\mathrm{correctly}\;\mathrm{predicted}\;\mathrm{targets}\;\mathrm{in}\;\mathrm{class}\;m\;/\;\mathrm{total}\;\mathrm{testing}\;\mathrm{of}\;\mathrm{class}\;m\right)\;\times \;100\%;m=1,2,\ldots ,M]$$

The fusion takes place using the K classification vectors with $c_{k}$ arranged into a K×M classification matrix C.

#### 3.3.1. Arrogance

Each network has a different classification output from the other networks. This type of fusion select the max per-element (for each target) classification output across all classifiers. In simple terms, the network that has the greatest confidence in its classification is accepted as the final output classification.

(15) $$V(C)=(\underset{k=1,\ldots ,K}{\max}C_{k1},\ldots ,\underset{k=1,\ldots ,K}{\max}C_{kM})$$

Then, the predicted class is computed using $\arg\max_{i=1,\ldots ,M}(V(C))$.

#### 3.3.2. Simple Voting 

This takes the highest confidence class for each $c_{k}$, and then selects the class by the most occurring class i. In other words, we compute a vector V of a selected class per classifier by

(16) $$V(C)=[\arg\underset{m=1,\ldots ,M}{\max}(c_{1}),\ldots ,\underset{m=1,\ldots ,M}{\arg\max(}c_{K})]$$

Then, the classification is simply mode(V(C))*.* A null classification is generated, if the majority of CNNs do not agree on a class. This is counted as a missed classification during evaluation.

#### 3.3.3. Accuracy Weighted Sum

This takes the classification matrix C and computes a weighted confidence across all classifiers for each class using the classification accuracy $p_{j}$ as the weights in the sum. The vector $p_{j}$ is computed as the per class, average cross-validation performance for each network, j∈[1,…N]. Let $C_{j}$ denote the *j*th row of the classification matrix, then

(17) $$V(C)=[p_{1}.C_{1}^{T},\ldots ,p_{M}.C_{M}^{T}]\;$$

The predicted class, i, is then $\arg\max_{i}(V(C))$*.* Intuitively, these weights are calculated from each network by its past performance measured from the cross-validation testing.

## 4. Methodology

**Figure 2:**
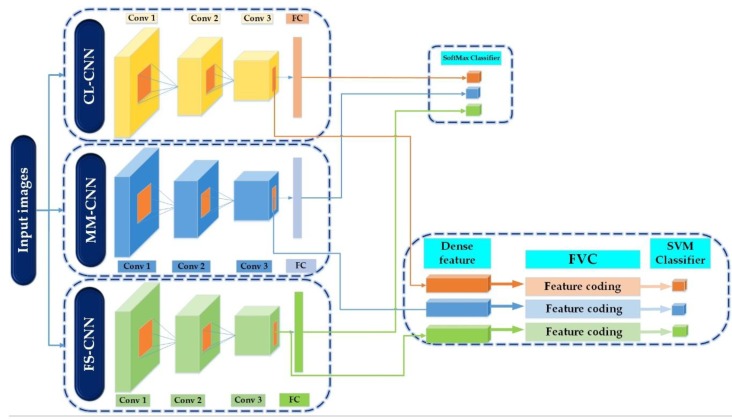
The framework of the proposed method.

**Figure 3:**
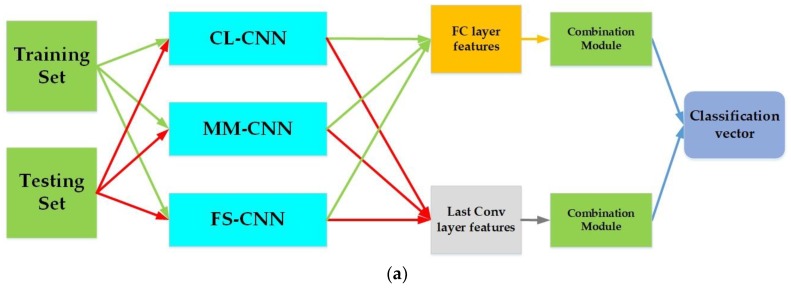

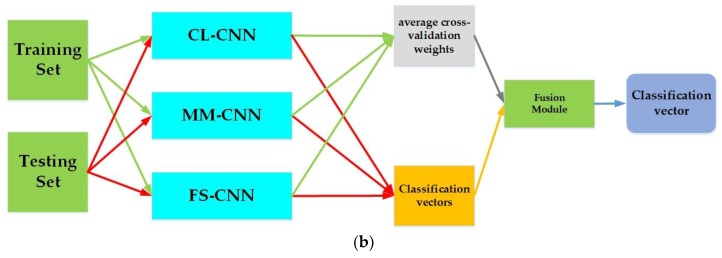
Final classification vector based on the three CNN architectures (**a**). Feature combination of the three CNN architectures (**b**) Fusion of the three CNN architectures.

For SAR image classification, several CNN architectures were proposed in the literature and have proven that the activations from high-level layers of CNNs can generate powerful feature representations with outstanding performance. However, two important points were noticed. First, the best classification accuracy for individual SAR classes is varied between the different CNN architecture and second, features extracted from lower-level layers, particularly the convolutional layers, lack sufficient study. These two reasons have encouraged us to propose different scenarios for utilizing CNN features for SAR images classification for the sake of investigating the effectiveness of features from the last convolutional layer and FC layer as well as the effect of combination and fusion different features and classifiers. Figure 2 and Figure 3 illustrate the framework of the proposed method.

The proposed method of SAR image classification is based on the following steps:

1. We use a set of different convolutional neural networks learning at different kernel sizes of convolution and pooling to produce high level invariant features;
2. The high-level features obtained from the FC layer are classified for each CNN model separately.
3. The features obtained from the last convolutional layer are classified after being encoded using the FVs coding.
4. A final feature vector from the FC layer is obtained by different combination of results of steps 2 and 3.
5. A final classification decision using different fusion methods based on results of steps 2 and 3 is obtained.

In this work, we have elected to apply the network topology presented in Section 3. Three different CNNs models at different kernel sizes of convolution and pooling were designed and trained on the MSTAR training set.

The choice of different parameters of each CNN model was mostly based on both previous types of research architecture [16,20,27,28,29] and experiments where we change the different parameters of CNN to obtain the architecture that achieves the best performances. We remark that several types of architecture can generate good SAR image representation. However, the best classification accuracy for individual classes is varied between the different forms of CNN architecture, which encourage us to exploit three kinds of CNN models with different convolution and pooling kernel size in each layer. We manage to have the same size of the FC layer in each architecture for reasons of simplicity in the two last steps of the proposed method, which are feature combination and the fusion. We chose the following appellation for the three architecture based on their respective convolution and pooling kernel sizes: CL-CNN, coarse grain with larger size, MM-CNN, middle grain with medium size, FS-CNN, fine grain with small size. The architecture of each network is given in Table 1. The convolution layer receives inputs from a local region of the input volume located in the small neighborhood of the previous layer, which is called the local receptive field. A typical convolutional layer has several feature maps. Weight vectors between different feature maps are different but all the units within one feature map share the same set of weights. Due to the use of local receptive fields and weight sharing, the number of free parameters to be learned is significantly reduced. BN layer helps in obtaining higher overall accuracy and faster learning. ReLu layer improves the networks by speeding up the training since it keeps the computation of the gradient very simple. Subsampling layer usually implemented as max-pooling layer further reduces feature dimension with translational invariance. FC layer is similar to classical neural networks computing a dot product between their input vector and their weight vector. The Softmax nonlinearity is utilized as the final output layer to deal with the multiclass classification issue.

Accuracy is calculated from each FC layer of the three CNN models using the SoftMax classifier, as well as from the dense features obtained from the last convolutional layers of each model, using the SVM classifier after being encoded by FVC. Figure 2 resumes the first three aforementioned steps of the proposed method.

Figure 3 resumes the last two above-mentioned steps of the proposed method. The combination module in Figure 3a could be either the concatenation method or the addition. In Figure 3b, the fusion module is one of the three aforementioned fusion methods in Section 3.3. The average cross-validation weights are needed only in the case where the fusion module is the Accuracy Weighted Sum. 

The results of each individual CNN model motivate us to use different techniques of combination and fusion. Since the CNN which produced the best classification accuracy for individual classes is varied between the different CNNs, this suggests that differences in the CNN architecture types may allow one network to consistently perform better on a subset of the SAR classes even though it underperforms, on average, across all the classes compared with another network. Consequently, feature combination and fusion of the CNNs information outputs as shown in Figure 3 should improve the overall robustness and accuracy of the result compared to a single CNN.

## 5. Experiment Results and Analysis

In our experiments, we implement different CNN models for feature extraction and classification and try different combinations of CNNs to improve the classification accuracy. Moreover, further treatment was applied using the FVs to improve the quality of the obtained features, followed by several combination and fusion methods. 

### 5.1. Experimental Setup

In this section, we investigate the representative power of CNNs features and evaluate the proposed models on real SAR image classification problem. The detailed experimental setup and numerous experiments with reasonable analysis are presented. We evaluate the effectiveness of CNN features on the following publicly available SAR dataset.

**Figure 4:**
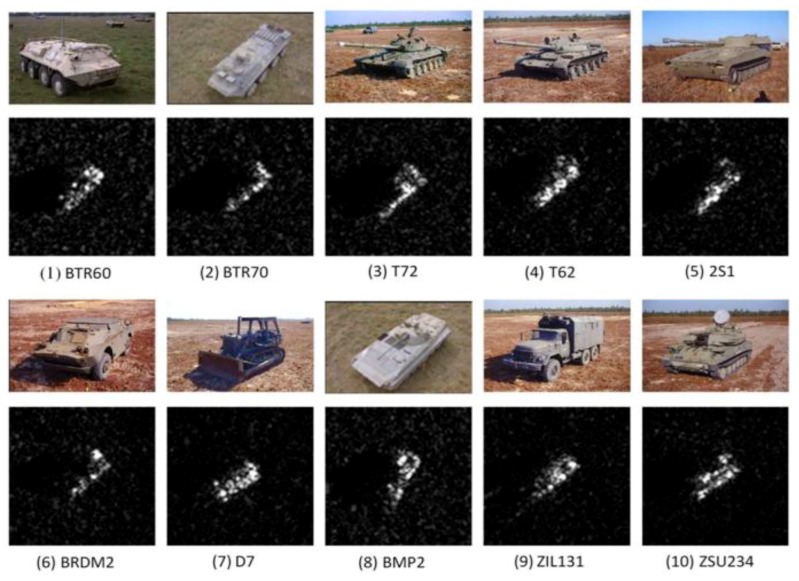
Examples of the ten class SAR targets.

The MSTAR benchmark data set is generally used to compare and test the performance of SAR-ATR algorithms. It was collected by the Sandia National Laboratory SAR platform [49]. Hundreds of thousands SAR images enclosing ground targets were collected, containing different target types, aspect angles, depression angles, serial number, and articulation. However, only a limited subset is publicly accessible on the website [50]. The publicly data set available consists of ten different classes of ground targets: (air defense unit: ZSU-234; armored personnel carrier: BMP-2, BRDM-2, BTR-60, and BTR-70; tank: T-62, T-72; rocket launcher: 2S1; truck: ZIL-131; bulldozer: D7). They were collected using an X-band SAR sensor, in a one feet resolution spotlight mode, with full aspect coverage. Each image has a size of around 128 by 128. The images are already centered with 0 °C to 360 °C degrees of orientation. Figure 4 depicts examples of SAR images of ten types of targets at similar aspect angles and their corresponding optical images. The data set is composed of 5172 image decomposed into 2747 training images and 2425 testing images. The distribution of training and testing data for the different targets is illustrated in Table 2.

Before introducing the experimental setup, it is worth noting that only the original number of images is used i.e., No data augmentation algorithm is applied. Each target with the same serial number in the training and testing sets differs in the azimuth and depression angle. Images for training are captured at a 17 ° depression angle, and images for testing are acquired at a 15 °C depression angle. We resize all images to 128×128 size where the target is in the center of the image. Also after this step, we have subtracted the mean from the images and divided it by the standard deviation of the images, in other words, we have normalized the data. 

### 5.2. Results of the First Set of Experiments

For the first set of experiments, all the CNN models introduced in Section 4 are evaluated and compared. The training subset is introduced as a cell array of image data, where the data in each cell have the same dimension (128×128), and then it is fed to the CNN catalog. Finally, the SAR images are classified into ten classes. The classification performances of the three different CNN models are shown in Table 3. The resulting high classification accuracies reveal the powerful ability of the chosen CNNs models.

**Figure 5:**
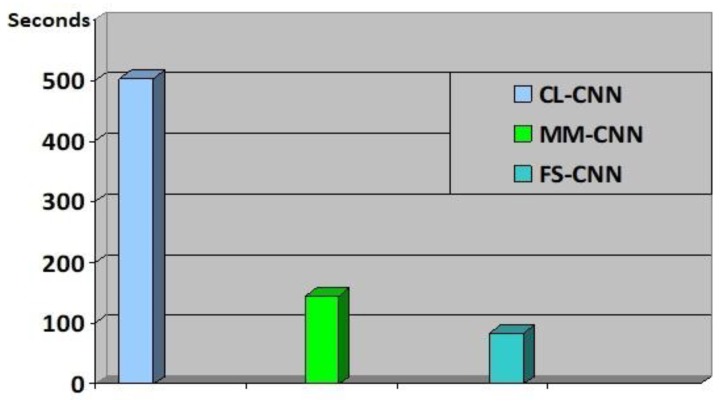
The time consumption of computing CNN activations with different CNN architectures for all SAR images in the MSTAR dataset.

We evaluate the time consumption (measured in terms of seconds) of training and testing all the three CNNs models all image in the MSTAR dataset, shown in Figure 5. As expected, CL-CNN has the biggest computational time consumption followed by MM-CNN and finally the FS-CNN. Although the three models have the same structure, they have very different computational time, due to the number of operations in each network. In our case, the size of the first pooling layer in each network has much more effects on the computation time than the other layers’ parameters. The bigger the size, the smaller the time consumption.

### 5.3. Results of the Second Set of Experiments

For the second set of experiments, we evaluate the global CNN features of the three CNN models. Dense features are extracted from the last convolutional layer and then encoded into global image features through one of the commonly used remote sensing feature coding approaches which is FVs coding. The dense features are L2-normalized prior to applying feature coding. We empirically set the number of Gaussian components in the GMM to 100. 

We randomly select samples of each class for training the SVM classifier. The classification accuracy is measured by $A=N_{c}/N_{t}$, where $N_{c}$ denotes the number of correctly classified samples in the testing samples and $N_{t}$ denotes the total number of testing samples. We evaluate the final classification performance with the average accuracy A over 50 runs. The public LIBLINEAR library [51] is used for SVM training and testing with the linear kernel. The open source library VLFeat is also used [52] for implementing the FVs coding. The overall performance of the IFK when using the three CNN models convolutional features from the last convolutional layer are shown in Table 4. The accuracies of IFK on MSTAR dataset on the three models exceed all the results of using FC features.

### 5.4. Combining Features from the FC Layer

In the first set of experiments, we generated the global image representations solely via the CNN models. We can take it a step further by combining the features computed by these three frameworks. We use two techniques of combination: (1) addition and (2) concatenation, to combine among the extracted features. For addition, the size of transformed features is the same. In the concatenation combination, the size of the features is multiplied by 3. Finally, SVM is used for classification. Here, we present some tentative combinations using the three different CNNs, and the results are shown in Table 5. We observe that on the MSTAR dataset, the combined features improve performance with approximately 1–2% gains compared to the separate cases.

### 5.5. Combining Features from the Last Convolutional Layer

In the second set of experiments, we generated the global image representations via the FVs after extracting the dense features from the last convolutional layer for each CNN model. We combine the features computed by these three frameworks by using the same techniques (addition and concatenation). Finally, SVM is used for classification. Some tentative combinations using the three different CNNs are shown in Table 6. 

It can be easily observed the combined features generated by the last convolutional layers improve performance with approximately 1–2% gains compared to the separate situations. So in both cases, the combination of features improves the accuracy performances even though the same structure was used in the three CNN models, which proves that differences in the CNN architecture allow one network to consistently perform better on a subset of the SAR images. In another words the gain would not be possible if the three CNN models classify the SAR images in the same way. Consequently, feature combination of the CNNs information improve the overall robustness and accuracy of the result compared to a single CNN.

### 5.6. Decision Fusion of the First and the Second Sets of Experiments Classifiers

After classification of the three CNN models separately by their individual classifiers for the two sets of experiments, decision fusion algorithms mentioned in Section 3.3 are used sequentially and the results of different combinations for fusion are summarized in Table 7 and Table 8. It is clear from the two tables that fusing the results of the second set of experiments (with FVC) outperforms those of the first set. The simple voting method achieved 98.39% classification accuracy on results of the second set of experiments when combining CL-CNN and FS-CNN. The arrogance method achieved the best accuracy, with a 98.60% classification. The accuracy weighted sum method achieved its best rate when fusing the three models with a 98.35% classification accuracy. Each method used for fusion outperformed the single best CNN baseline result.

## 6. Conclusions

In this paper, we have investigated the SAR images classification task on the MSTAR dataset using the activations of different CNN models. In order to obtain strong global image representations for classification, various sets of experiments are proposed by directly extracting features from the FC layers and encoding dense features from convolutional layers. We evaluate our methods on the public MSTAR dataset and achieve remarkable overall classification accuracies of 98.60% for the ten-classification task. As expected, our method obtained remarkable results competitive with the state-of-the-art methods. Even though we do not apply any data augmentation strategies in our method in contrast with the methods that also explore CNNs, our method is only 0.4% worse than the best method that elaborately uses transfer learning from SAR images scenes from the CNN, and better than methods which use pre-trained CNN models. Furthermore, combining and fusing scenarios improves performance by about 2–3%. These impressive results demonstrate that not only the features of FC layers but also dense convolutional features of CNN are discriminative and powerful image representations. In future studies, we plan to investigate more sophisticated strategies for encoding the dense CNN features to improve the invariance of representations.

## Figures and Tables

**Table 1:** The architecture of the three CNN networks.

	CL-CNN			MM-CNN			FS-CNN		
	Image Size	Feature Maps	Kernel Size	Image Size	Feature Maps	Kernel Size	Image Size	Feature Maps	Kernel Size
**Input layer**	128 × 128	1	-	128 × 128	1	-	128 × 128	1	-
**Convolutional**	104 × 104	8	25 × 25	111 × 111	8	18 × 18	120 × 120	8	9 × 9
**BN**	104 × 104	8	-	111 × 111	8	-	120 × 120	8	-
**ReLu**	104 × 104	8	-	111 × 111	8	-	120 × 120	8	-
**pooling**	52 × 52	8	2 × 2	37 × 37	8	3 × 3	30 × 30	8	4 × 4
**Convolutional**	40 × 40	16	13 × 13	32 × 32	16	6 × 6	26 × 26	16	5 × 5
**BN**	40 × 40	16	-	32 × 32	16	-	26 × 26	16	-
**ReLu**	40 × 40	16	-	32 × 32	16	-	26 × 26	16	-
**pooling**	20 × 20	16	2 × 2	16 × 16	16	2 × 2	13 × 13	16	2 × 2
**Convolutional**	12 × 12	32	9 × 9	12 × 12	32	5 × 5	12 × 12	32	2 × 2
**BN**	12 × 12	32	-	12 × 12	32	-	12 × 12	32	-
**ReLu**	12 × 12	32	-	12 × 12	32	-	12 × 12	32	-
**pooling**	6 × 6	32	2 × 2	6 × 6	32	2 × 2	6 × 6	32	2 × 2
**FC**	1	1152	-	1	1152	-	1	1152	-
**Output**	1	10	-	1	10	-	1	10	-

**Table 2:** Type and sample number of training and testing set.

Target	ZIL131	ZSU_234	T72	T62	D7	2S1	BMP2	BRDM2	BTR_60	BTR_70
**Training set**	299	299	232	299	299	299	233	298	256	233
**Testing set**	274	274	196	273	274	274	195	274	195	196

**Table 3:** Type and sample number of training and testing set.

Model	Classification Accuracy (%)
CL-CNN	95.95
MM-CNN	96.12
FS-CNN	96.17

**Table 4:** Overall Classification Accuracy of FVs Coding Method Using Different CNN architecture.

Model	Classification Accuracy (%)
CL-CNN	97.29
MM-CNN	97.18
FS-CNN	96.77

**Table 5:** Performance of Combining Features Generated by the FC layer.

Combination			Type of Combination	Accuracy Performance (%)
CL-CNN	MM-CNN		Addition	98.45
CL-CNN		FS-CNN	Addition	98.31
	MM-CNN	FS-CNN	Addition	98.56
CL-CNN	MM-CNN		Concatenation	98.37
CL-CNN		FS-CNN	Concatenation	98.42
	MM-CNN	FS-CNN	Concatenation	98.53
CL-CNN	MM-CNN	FS-CNN	Addition	98.27
CL-CNN	MM-CNN	FS-CNN	Concatenation	98.41

**Table 6:** Performance of Combining Features Generated by the last convolutional layers.

Combination			Type of Combination	Accuracy Performance (%)
CL-CNN	MM-CNN		Addition	98.45
CL-CNN		FS-CNN	Addition	98.31
	MM-CNN	FS-CNN	Addition	98.56
CL-CNN	MM-CNN		Concatenation	98.34
CL-CNN		FS-CNN	Concatenation	98.42
	MM-CNN	FS-CNN	Concatenation	98.53
CL-CNN	MM-CNN	FS-CNN	Addition	98.42
CL-CNN	MM-CNN	FS-CNN	Concatenation	98.53

**Table 7:** Performance of fusing classifiers outputs from the first set of experiments.

Fusion Combination			Type of Fusion	Accuracy Performance (%)
CL-CNN	MM-CNN		Arrogance	97.48
CL-CNN	MM-CNN		Simple voting	97.53
CL-CNN	MM-CNN		Weighted Sum	97.40
CL-CNN		FS-CNN	Arrogance	98.19
CL-CNN		FS-CNN	Simple voting	97.57
CL-CNN		FS-CNN	Weighted Sum	98.19
	MM-CNN	FS-CNN	Arrogance	97.94
	MM-CNN	FS-CNN	Simple voting	98.02
	MM-CNN	FS-CNN	Weighted Sum	97.90
CL-CNN	MM-CNN	FS-CNN	Arrogance	98.19
CL-CNN	MM-CNN	FS-CNN	Simple voting	97.98
CL-CNN	MM-CNN	FS-CNN	Weighted Sum	98.14

**Table 8:** Performance of fusing classifiers outputs from the second set of experiments.

Fusion Combination			Type of fusion	Accuracy Performance (%)
CL-CNN	MM-CNN		Arrogance	98.60
CL-CNN	MM-CNN		Simple voting	98.14
CL-CNN	MM-CNN		Weighted Sum	98.23
CL-CNN		FS-CNN	Arrogance	98.60
CL-CNN		FS-CNN	Simple voting	98.39
CL-CNN		FS-CNN	Weighted Sum	98.31
	MM-CNN	FS-CNN	Arrogance	98.60
	MM-CNN	FS-CNN	Simple voting	98.27
	MM-CNN	FS-CNN	Weighted Sum	98.39
CL-CNN	MM-CNN	FS-CNN	Arrogance	98.60
CL-CNN	MM-CNN	FS-CNN	Simple voting	98.27
CL-CNN	MM-CNN	FS-CNN	Weighted Sum	98.35
